# Mice with an autosomal dominant Charcot-Marie-Tooth type 2O disease mutation in both dynein alleles display severe moto-sensory phenotypes

**DOI:** 10.1038/s41598-019-48431-7

**Published:** 2019-08-19

**Authors:** Swaran Nandini, Jami L. Conley Calderon, Thywill T. Sabblah, Rachal Love, Linda E. King, Stephen J. King

**Affiliations:** Burnett School of Biomedical Sciences, College of Medicine, University of Central Florida, Orlando, FL 32827 USA

**Keywords:** Neuromuscular disease, Mechanisms of disease, Peripheral neuropathies

## Abstract

Charcot-Marie-Tooth disease (CMT) is the most common peripheral neuromuscular disorder worldwide. The axonal degeneration in CMT causes distal muscle weakness and atrophy, resulting in gait problems and difficulties with basic motor coordination skills. A mutation in the cytoplasmic dynein heavy chain (DHC) gene was discovered to cause an autosomal dominant form of the disease designated Charcot-Marie-Tooth type 2O disease (CMT2O) in 2011. The mutation is a single amino acid change of histidine into arginine at amino acid 306 (H306R) in DHC. We previously generated a knock-in mouse carrying the corresponding CMT2O mutation (H304R) and examined the heterozygous H304R/+offspring in a variety of motor skills and histological assays. Here we report the initial characterization of the homozygous H304R/R mouse, which is the first homozygous mutant DHC mouse to survive past the neonatal stage. We show that H304R/R mice have significantly more severe disease symptoms than the heterozygous H304R/+mice. The H304R/R mice have significant defects in motor skills, including grip strength, motor coordination, and gait and also related defects in neuromuscular junction architecture. Furthermore, the mice have defects in sensation, another aspect of CMT disease. Our results show that the H304R/+ and H304R/R mice will be important models for studying the onset and progression of both heterozygous and homozygous CMT disease alleles.

## Introduction

Cytoplasmic dynein is a microtubule-based minus-end directed motor complex that plays critical roles in a variety of cellular functions^1^. Dynein is the predominant minus end directed microtubule motor, whereas there are dozens of plus-end directed kinesin motors that have more specialized roles in cells. It is not surprising that the *Dync1h1* gene encoding the dynein heavy chain (DHC) polypeptide is an essential gene, as this gene encodes the ~4600 amino acid polypeptide that is present as a dimer in each functional cytoplasmic dynein molecule. Homozygous DHC−/− null mice do not develop beyond the blastula stage due to severe defects in intracellular transport, especially affecting maintenance of Golgi structure and function^2^. That same report showed that dynein is not affected by genetic haploinsufficiency as DHC+/− mice developed normally and had no reported defects^2^.

With new capabilities to screen and identify mutations in affected populations, there has been a rapid identification of mutations within the *Dync1h1* gene that appear to be the causative agents in a variety of neurological disorders. The first such report was the identification of a missense mutation changing histidine 306 to arginine (H306R) in the *Dync1h1* gene, leading to an inheritable autosomal dominant form of Charcot-Marie-Tooth disease (CMT) found in 23 members of an extended four-generation family^3^. The particular isoform of CMT caused by the H306R dynein mutation is classified as Charcot-Marie-Tooth disease type 2 O (CMT2O), and is an autosomal dominant disease mutation that is grouped with other CMT type 2 mutations (CMT2) whose gene products have axonal functions in nerve cells. A second report identified the identical *Dync1h1* mutation as the causative mutation in a family with three members affected by spinal muscular atrophy with lower extremity predominance (SMA-LED)^4^. Both CMT2O and SMA-LED patients display some common neuropathic symptoms such as muscle weakness and wasting (primarily in legs), mild to severe loss of motor functions, motor milestone developmental delays, problems in walking, and skeletal defects such as joint contractures and foot deformities. The main difference between CMT and SMA-LED diagnoses is the presence of sensory defects in CMT patients but not in SMA-LED patients.

In an effort to study the onset and progression of CMT type 2 caused by a cytoplasmic dynein mutation, we previously developed a knock-in mouse carrying a H304R mutation in the *Dync1h1* cytoplasmic dynein heavy chain gene that corresponds to the H306R mutation in humans^5^. In that study, we examined the effect of the H304R mutation in heterozygous H304R/+ mice, which correlates to the autosomal dominant status of the human H306R/+ CMT2O mutation. We determined that H304R/+ mice exhibited a range of motor skill defects that are commonly found in patients with CMT disease. We also determined that H304R/+ mice have alterations in the structure and organization of the neuromuscular junctions (NMJs) in lower limb skeletal muscle.

However, the phenotypes we observed in that study were not as severe as the phenotypes observed in previous studies with the three other heterozygous DHC mutant mouse lines in existence: *Loa*/+, *Cra*/+, and *Swl*/+. The autosomal dominant mutations in *Loa* (F580Y), *Cra* (Y1055C), and *Swl* ([GIVT]1040[A]) mice do not correspond to human mutations as they were generated by mutagenesis and screened for locomotor defects^6–8^. The heterozygous *Loa*/+, *Cra*/+ and *Swl*/+ mice had normal lifespans and exhibited altered motor phenotypes of hind limb clenching, gait abnormalities, and reduced hind limb strength^9,10^. Heterozygous *Loa*/+ and *Swl*/+ mice also showed an absence of the H-reflex, proprioceptive sensory defects, and a loss of neurons in their lumbar dorsal root ganglia^9^. For *Loa/Loa*, *Cra/Cra*, and *Swl/Swl* mice, researchers determined that the homozygous mutations were embryonic or neonatal lethal^9,11^.

It is important to remember that functional cytoplasmic dynein motors in cells contain a dimer of DHC polypeptides plus other dynein polypeptides. Therefore, dynein motor molecules in heterozygous mutant animals are expected to contain DHCs in the following ratio: a quarter of the dynein motors will have a DHC dimer with two wild type monomers, a quarter will have a DHC dimer with two mutant monomers, and half will have a DHC dimer containing one wild type and one mutant monomer. The presence of these three categories of dynein motors within heterozygous animals may be a part of the basis for disease onset and progression, but certainly complicate the understanding of what an individual mutation may be doing to the population of dynein motor molecules inside cells.

To better examine how the H304R mutation may lead to the CMT2O disease state, in this study we generated a homozygous H304R/R mouse model in which both *Dync1h1* alleles harbor the autosomal dominant H304R mutation, meaning that there would exist a homogeneous population of dynein dimers each containing two mutant DHC monomers. The homozygous H304R/R mice are not models for loss of function studies as each mutant allele is instead autosomal dominant. Here we present our initial examination of the homozygous H304R/R mice and show they have defects in their motor skills, neuromuscular junctions, sensation, and gait that are more severe than those we determined for heterozygous H304R/+ mice. The study of a model system with homozygous autosomal dominant alleles is relevant to DHC-related human disease as there has been a report of an autosomal dominant human DHC mutation (R399G) in a homozygous offspring. The homozygous R399G/G human offspring had much more severe symptoms than those of the heterozygous R399G/+ parents^12^, similar to what we report here.

## Results

### Homozygous H304R/R mice have a normal lifespan

In this study we successfully bred and characterized autosomal dominant DHC mutant mice carrying the H304R mutation in both chromosomal copies (Suppl. Fig. 1). The homozygous H304R/R mutation does not alter the amount of dynein motor present in brain tissue, as determined by Western blot assay (Suppl. Fig. 2). Homozygous H304R/R mice had a normal life span unlike the previously reported dynein mutant mice homozygous pups (*Loa/Loa*, *Cra/Cra*, *Swl/Swl*)^8,11^. Those other DHC homozygous mutant mice were unable to survive past 24 hours after birth due to their inability to feed. Additionally, homozygous H304R/R male and female mice were also able to successfully reproduce with each other to yield homozygous pups. Visually, some H304R/R mice appeared to be smaller than their wild type and H304R/+ littermates. However, the overall H304R/R mice population did not have any significant bodyweight differences as compared to wild type and H304R/+ littermates. We found no change from the expected ratio of 50% male and 50% female mice for heterozygous H304R/+ or homozygous H304R/R offspring in litters.

### Homozygous H304R/R mice display atypical tail suspension reflex

**Figure 1:**
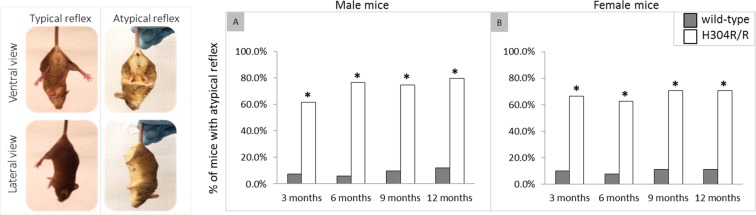
Tail suspension phenotype in wild type and H304R/R mutant mice. Left: The typical tail suspension reflex is hind limbs splaying away from the body. The atypical reflex is hind limbs clenching together closer to the body. Right: Male (**A**) and female (**B**) atypical tail suspension reflexes were scored per 3-month long time intervals and were statistically compared between the wild type and H304R/R mice using Fisher’s Exact test (two-tailed distribution, *p* < 0.05). The wild-type data was generated and previously reported in Sabblah *et al*.^5^.

A tail suspension test was performed to quantify the neuropathological phenotype of the homozygous H304R/R mice (Fig. 1). We observed that a significant majority (60–80%) of male H304R/R mice displayed the atypical reflex of clenching its hind limbs when gently suspended by its tail as compared to the wild-type mice at all ages (*p* < 0.05). Similarly, a significant majority (65–70%) of female H304R/R mice displayed the atypical tail suspension reflex as compared to the wild-type mice (*p* < 0.05). Both male and female H304R/R mice exhibited the atypical tail suspension reflex from an early age and throughout their lives (Suppl. Table 1).

### Homozygous H304R/R mice have reduced grip strength

**Figure 2:**
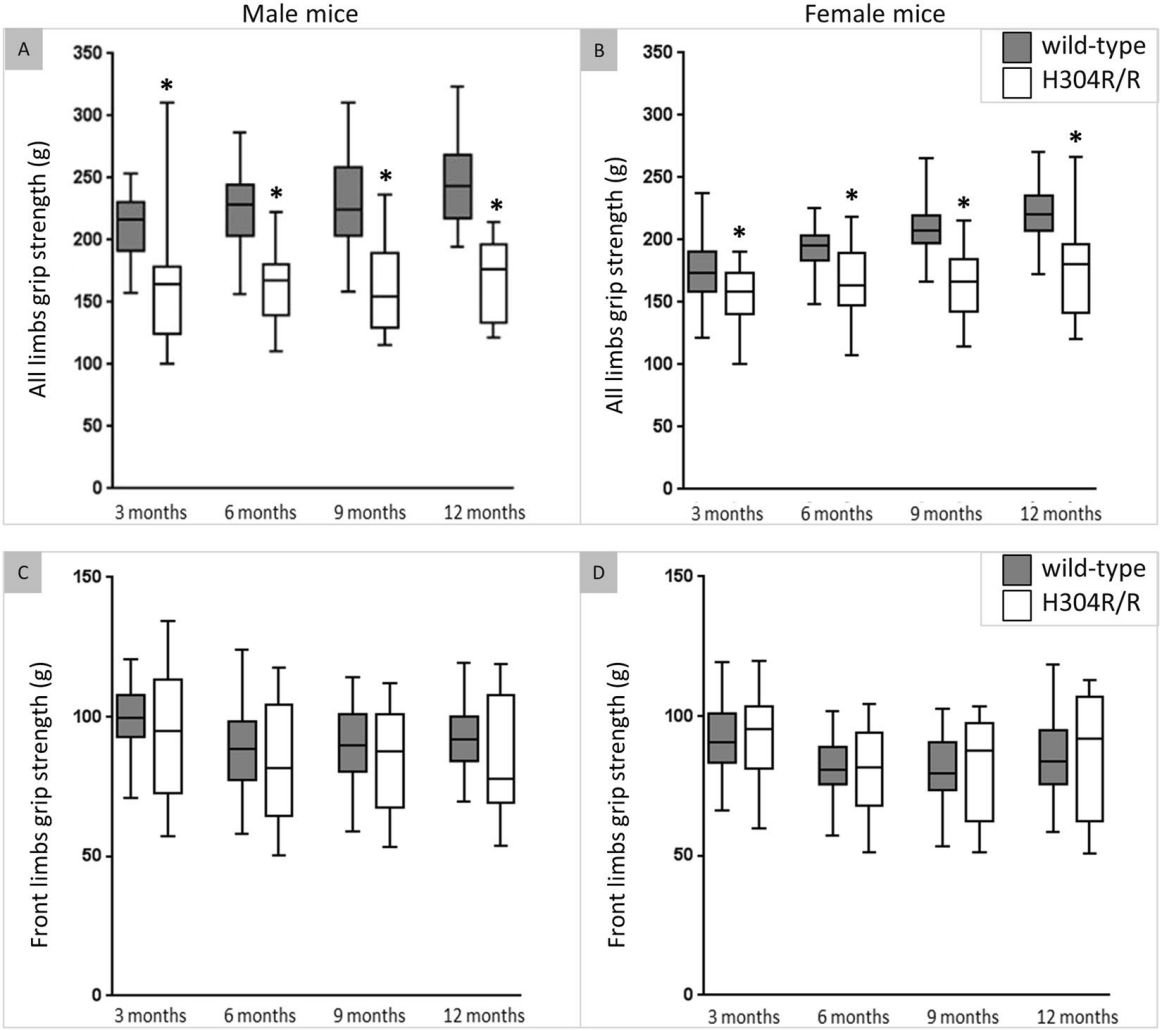
Grip strength analyses in wild type and H304R/R mutant mice. The top panels show all limbs grip strength (grams) in male (**A**) and female (**B**) H304R/R mice. The bottom panels show front limbs grip strength (grams) in male (**C**) and female (**D**) H304R/R mice. The box plot represents the 25^th^ to 75^th^ percentile of the data points with a middle line at the median and the whiskers ranging from the smallest to the largest value. The grip strength data was collected and averaged per 3-month time interval for each test mouse, and was compared between the wild-type (in dark gray) and H304R/R mice (in white) using the Welch’s t-test (two-tailed distribution, *p < 0.05). The wild-type data was generated and previously reported in Sabblah *et al*.^5^.

We performed a standard grip test to measure the limb muscle strength in homozygous H304R/R mice (Fig. 2). Male H304R/R mice showed a statistically significant reduction (23–31%) in strength in all four limbs as compared to the wild-type mice at all ages (*p* < 0.05) (Suppl. Table 2). Female H304R/R mice also showed a similar trend, displaying a statistically significant reduction (12–22%) in strength in all four limbs as compared to the female wild-type mice at all ages (*p* < 0.05) (Suppl. Table 3). Both male and female H304R/R mice did not show any significant reductions in their front limb grip strengths as compared to the wild-type control mice. Our data showing grip strength deficiencies only in the all limbs condition but not in the front limbs condition indicates that there is hind limb muscle weaknesses that contributes to the overall reduction in grip strength in mutant H304R/R mice. The H304R/R mice grip strength data correlates with the human CMT2O clinical symptoms where patients display muscular weaknesses primarily in their lower limbs.

### Homozygous H304R/R mice have deficits in the rotarod test performance

**Figure 3:**
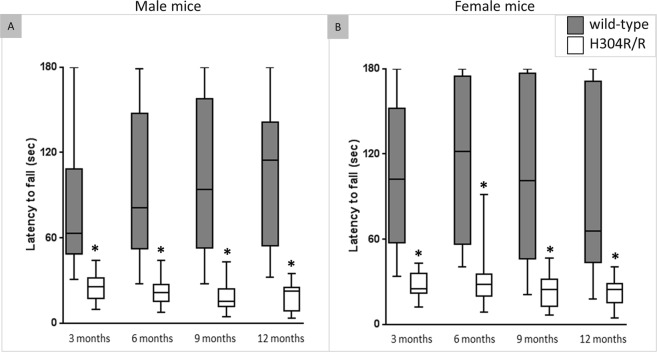
Rotarod performance analyses in wild type and H304R/R mutant mice. The graph shows the latency to fall (seconds) from a rotating rod in male (**A**) and female (**B**) mice. The box plot represents the 25^th^ to 75^th^ percentile of the data points with a middle line at the median and the whiskers ranging from the smallest to the largest value. The rotarod data was collected and averaged per 3-month time interval for each test mouse, and was compared between the wild-type (in dark gray) and H304R/R (in white) mice using the Welch’s t-test (two-tailed distribution, *p < 0.05). The wild-type data was generated and previously reported in Sabblah *et al*.^5^.

Rotarod tests were performed to assess the motor balance and motor coordination in homozygous H304R/R mice (Fig. 3). Male H304R/R mice showed a reduced latency to fall as compared to the male wild-type mice at all ages (*p* < 0.05) (Suppl. Table 2). Similarly, female H304R/R mice showed a decreased latency to fall as compared to the wild-type mice at all ages (*p* < 0.05) (Suppl. Table 3). Overall, there was about 65% reduction in the latency to fall in both male and female H304R/R mice as compared to the wild-type mice. The significant decline in the ability to sustain movement on the rotating rod indicates a lack of motor coordination in both male and female H304R/R mice.

### Gait analysis of the H304R/+ and H304R/R mouse strains

Many H304R/R mice appeared to exhibit an altered gait when moving inside their cage as compared to their littermates of other genotypes. We performed a gait analysis on the H304R/+ and H304R/R mice, alongside wild-type controls, to determine if they displayed gait phenotypes consistent with CMT. Human patients with CMT, including CMT2O, present with many symptoms of varying severity, but patients frequently display an altered gait. Gait can be broken down into two main phases: stance and swing. The stance phase takes place when the foot is in contact with the ground; the swing phase occurs when the foot is lifted and no longer in contact with the ground. The stance phase can be further divided into the brake and propulsion subphases. The brake subphase occurs when the foot initially makes contact with the ground, whereas the propulsion subphase is when the subject propels forward and the foot pushes against the ground while still making contact. A study of humans with CMT1A previously showed reduced propulsion in children and adolescents^13^. No gait studies with CMT2 patients have been reported.

#### H304R/R mice display a decreased stride length and an increased stride frequency

**Figure 4:**
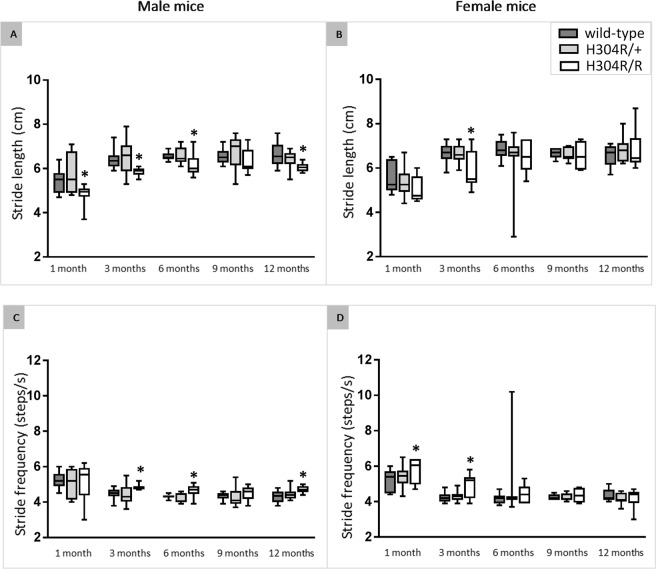
Analysis of hindlimb stride length and frequency in wild type, H304R/+, and H304R/R mice. The data show the average stride length for male (**A**) and female (**B**) mice as well as stride frequency of male (**C**) and female (**D**) mice tested at a constant belt speed of 28 cm/s. The box plot represents the 25^th^ to 75^th^ percentile of the data points with a middle line at the median and the whiskers ranging from the smallest to the largest value. The H304R/+ (in light gray) and H304R/R (in white) data were compared to the wild-type data (in dark gray) using one-way ANOVA (Tukey’s multiple comparisons test, *p < 0.05).

Male and female H304R/+ mice showed no statistically significant difference in stride length or stride frequency compared to wild-type mice. However, male H304R/R mice showed a significant decrease (*p* < 0.05) in their hind limb stride length compared to wild-type mice at 1,3,6, and 12 months of age when running at a constant speed of 28 cm/s (Fig. 4, Suppl. Table 4). Female H304R/R mice also displayed a significant decrease (*p* < 0.05) in their hind limb stride length compared to wild-type controls, but only at the 3-month time point (Fig. 4, Suppl. Table 4). As expected, the decreased stride length resulted in an increased hind limb stride frequency when mice were tested at a constant belt speed. Male H304R/R mice displayed a significant increase (*p* < 0.05) in stride frequency at 3, 6, and 12 months of age compared to wild-type controls, and female H304R/R mice showed a significant increase (*p* < 0.05) at 1 and 3 months of age (Fig. 4, Suppl. Table 4). These data parallel our previously published motor skills assessments showing mutant males display a more pronounced phenotype than females^5^.

#### H304R/+ and H304R/R mice exhibit differences in the subphases of gait

**Figure 5:**
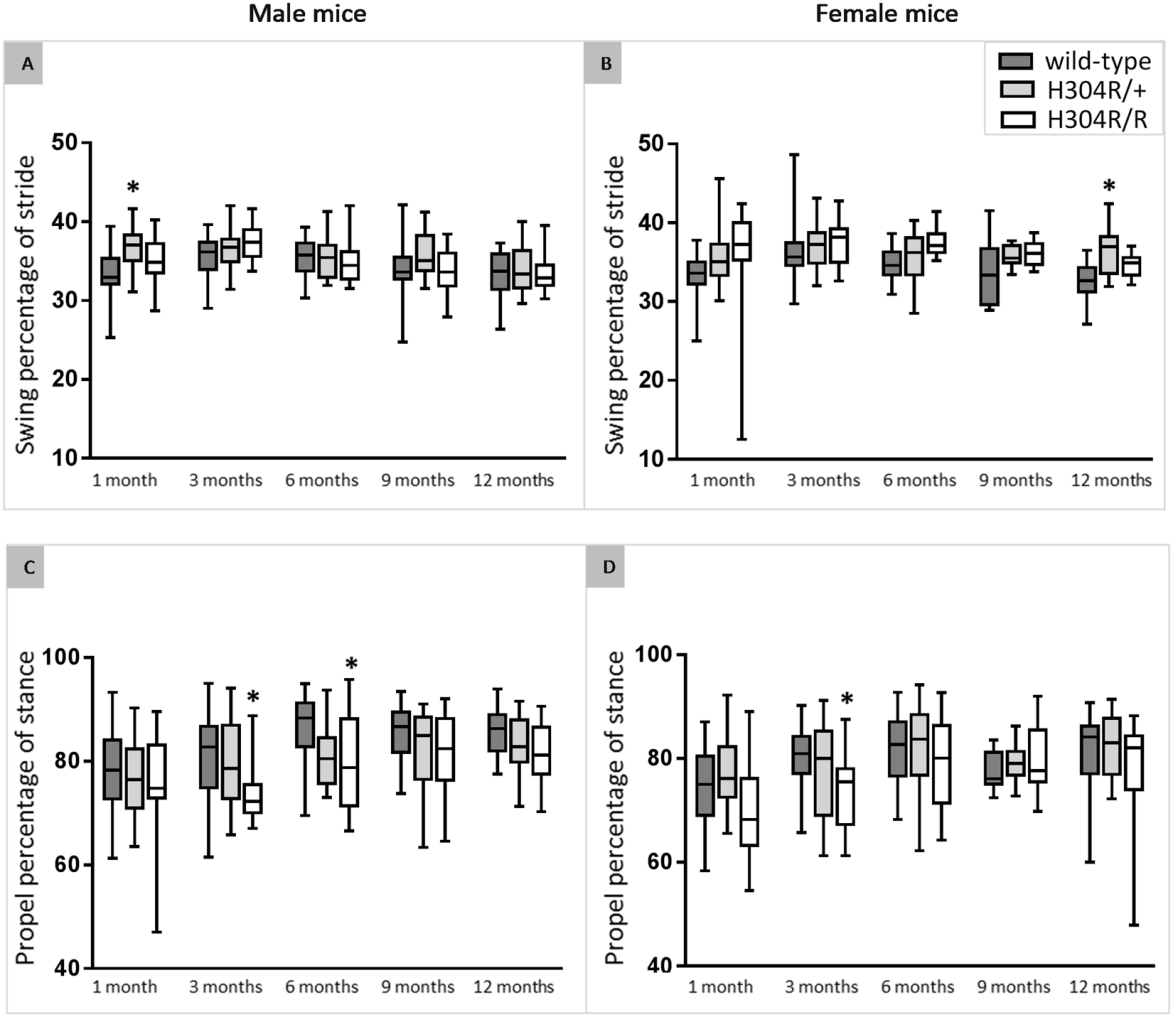
Analysis of percentage of time spent in various portions of the stride. The top data show the percentage of the stride spent in the swing phase in the hind limbs of male (**A**) and female (**B**) mice tested over a period of 12 months. The bottom data show the percentage of the stance phase spent propelling rather than braking in the hind limbs of male (**C**) and female (**D**) mice tested over a period of 12 months. The box plot represents the 25^th^ to 75^th^ percentile of the data points with a middle line at the median and the whiskers ranging from the smallest to the largest value. The H304R/+ (in light gray) and H304R/R (in white) data were compared to the wild-type data (in dark gray) using one-way ANOVA (Tukey’s multiple comparisons test, *p < 0.05).

Male H304R/+ mice showed a significant increase (*p* < 0.05) in the proportion of time spent in the swing phase of gait and less time in the stance phase compared to wild-type control mice at 1 month of age. Female H304R/+ mice also spent significantly more time in the swing phase at the 12-month time point (*p* < 0.05). Male and female H304R/+ mice exhibited no significant difference in the propulsion subphase, as a percentage of the stance phase (Fig. 5). Male H304R/R showed no difference in the proportion of stance and swing phases, but they displayed a significant decrease (*p* < 0.05) in the propulsion subphase at 3 and 6 months of age (Fig. 5A). Similarly, female H304R/R mice showed no change in the swing and stance phase proportions, but they displayed a reduction (*p* < 0.05) in the propulsion subphase at 3 months of age (Fig. 5B). This reduction in propulsion is consistent with gait analyses of children with CMT1A, in which 29% of the children tested at their natural walking speed showed reduced propulsion during the push-off subphase of their gait^13^.

#### H304R/+ and H304R/R mice show differences in paw angle

**Figure 6:**
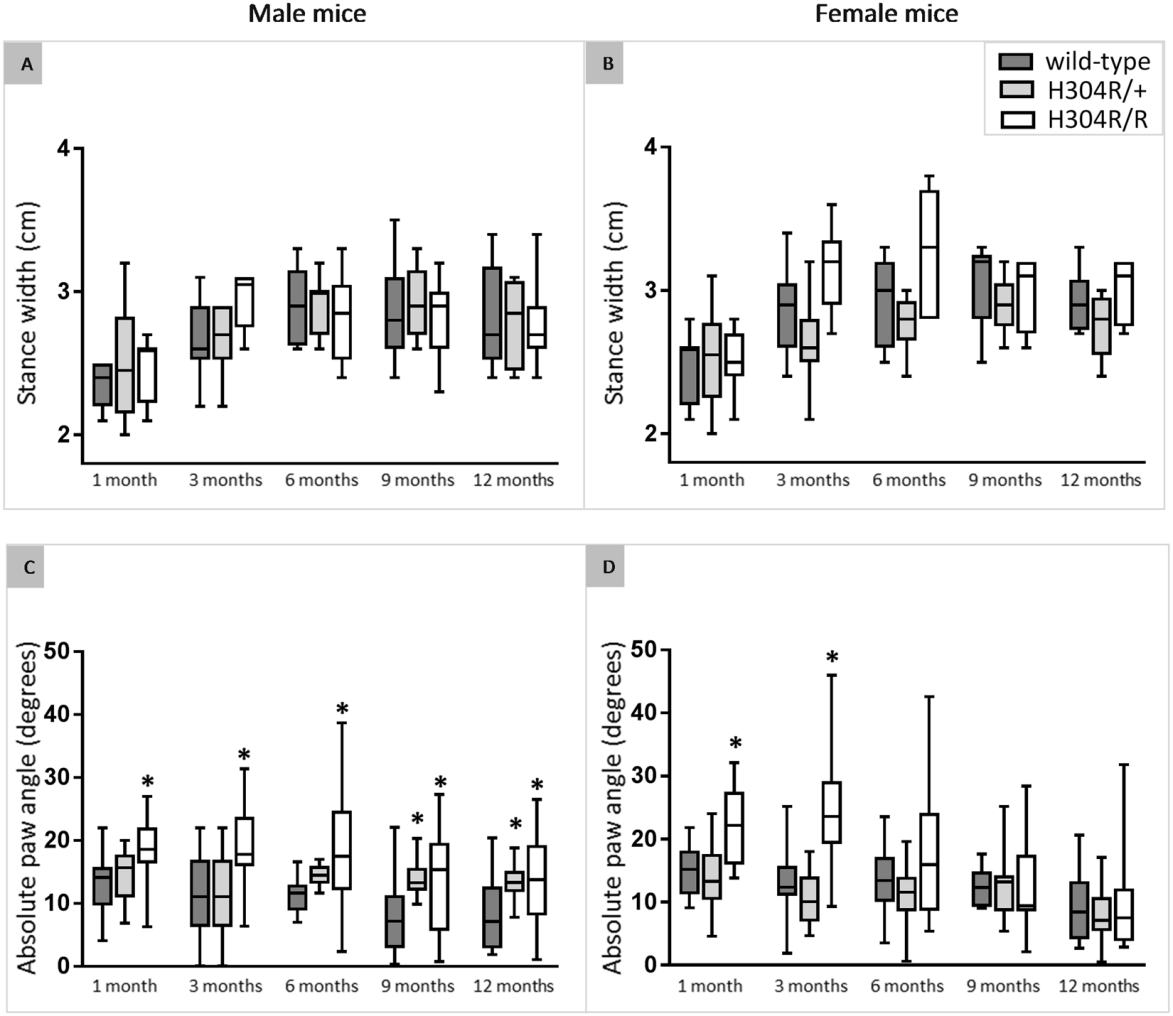
Analysis of stance width and paw angle in wild type, H304R/+, and H304R/R mice. The top data show the distance between the hind limbs during the stance phase of male (**A**) and female (**B**) mice tested up to 12 months of age. The bottom data show the absolute value of the paw angle during the stance phase of male (**C**) and female (**D**) mice tested up to 12 months of age. The box plot represents the 25^th^ to 75^th^ percentile of the data points with a middle line at the median and the whiskers ranging from the smallest to the largest value. The H304R/+ (in light gray) and H304R/R (in white) data were compared to the wild type data (in dark gray) using one-way ANOVA (Tukey’s multiple comparisons test, *p < 0.05).

Although H304R/+ and H304R/R mice display no differences in their stance width when running on the treadmill (Fig. 6A,B), they exhibit significant differences in paw angle compared to wild-type mice. Male H304R/+ mice showed a significant increase (*p* < 0.05) in paw angle at 9 and 12 months of age, showing that aged heterozygous mice rotate their paws outward during the stance phase (Fig. 6C). Female H304R/+ mice showed no difference in paw angle compared to wild-type controls (Fig. 6D). However, both male and female H304R/R mice showed increases in paw angle compared to wild-type controls. Male H304R/R mice showed a statistically significant increase (*p* < 0.05) in paw angle at all measured time points from 1 to 12 months of age (Fig. 6C). Female H304R/R mice showed a significant increase (*p* < 0.05) in paw angle at 1 and 3 months of age (Fig. 6D). These data together indicate that while the mice carrying the H304R mutation walk with a typical distance between their hind limbs, they angle their paws outward due to disease-related skeletal differences or potential compensation for hind limb muscle weakness^14,15^.

### Denervation at the neuromuscular junction

The neuromuscular junction (NMJ) is a known pathological target in many neuromuscular disorders as well as in some peripheral nerve disorders^16,17^. The nerves play essential roles in the set up and maintenance of the peripheral synaptic apparatus and therefore any form of pathology in the nerve can ultimately affect the structure and stability of the NMJ. We previously examined H304R/+ heterozygous dynein mice and determined that reduced innervation occurred at 9 and 12 months when compared with wild type mice^5^.

**Figure 7:**
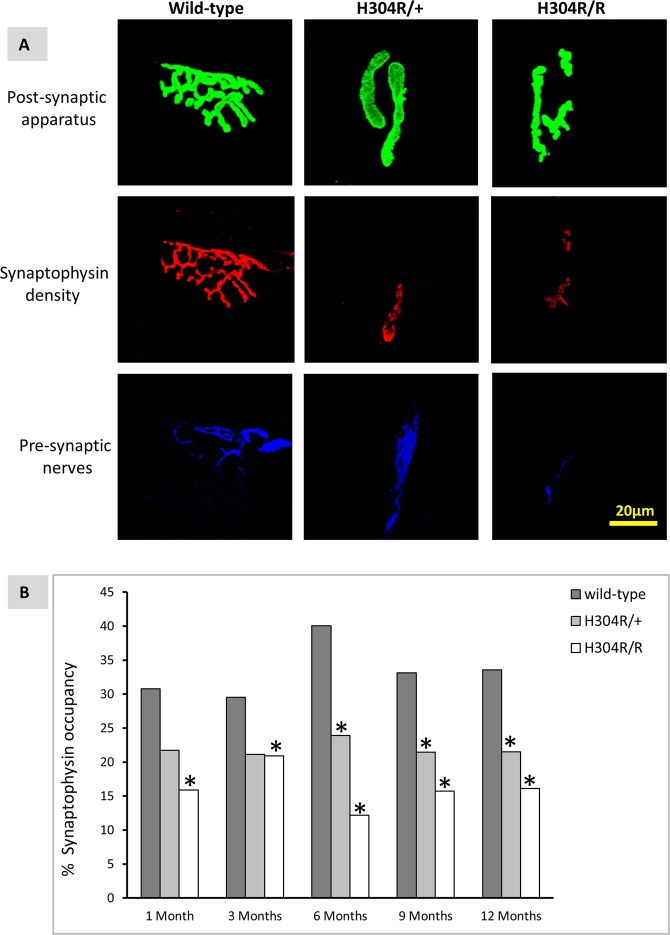
Innervation defects at the gastrocnemius neuromuscular junction (NMJ). Representative images from 12 months old mice showing post-synaptic acetylcholine receptors (AChR) stained with alpha-bungarotoxin (green), synaptophysin density (red) and pre-synaptic nerves stained with neurofilament (blue). (**A**) Percentage of synaptophysin occupancy (**B**) was computed as follows: (synaptophysin area/AChR area) x100 for the wild type (in dark gray), H304R/+ (in light gray), H304R/R (in white) mice. Statistical analysis was carried out using one-way ANOVA, (Tukey’s multiple comparisons test, *p < 0.05).

Here we examined innervation at the end-plate through neurofilament staining of nerve terminals in male H304R/R gastrocnemius muscle sections. Confocal microscopy revealed that there were multiple NMJs where nerves were completely absent. After quantitative analysis, we identified differences in the number of innervated NMJs and non-innervated NMJs between the homozygous and wild type mice at multiple time points (Fig. 7). The pronounced innervation defects in homozygous mice represents one more feature that distinguishes the homozygous H304R/R model as more severe than the heterozygous H304R/+ mouse model^5^.

We extended these results by asking whether the H304R mutation affects synaptic vesicle density in nerve terminals. We utilized the synaptic vesicle marker synaptophysin and determined its density at the NMJ^18^. Synaptic vesicle concentration could provide some insight into molecular motor function, as both dynein and kinesin are reported to colocalize with synaptic vesicles during nerve injury^19^. We compared synaptophysin vesicle density between wild-type, H304R/+, and H304R/R mice by calculating the percentage of coverage of synaptophysin staining compared to the area of AChR staining. In heterozygote H304R/+ mice, there were no statistically significant differences in NMJ synaptic vesicle density at early time points compared to wild type NMJs. However, significant differences were observed in H304R/+ NMJs from mice that were 6 months and 12 months of age (Fig. 7, Suppl. Table 5). In H304R/R mice, synaptic vesicle density at NMJs was consistently lower than wild-type and H304R/+ mice. The differences between wild-type and homozygous were significant at all time points examined.

### Neuromuscular deficits in the H304R/R homozygous mice

**Figure 8:**
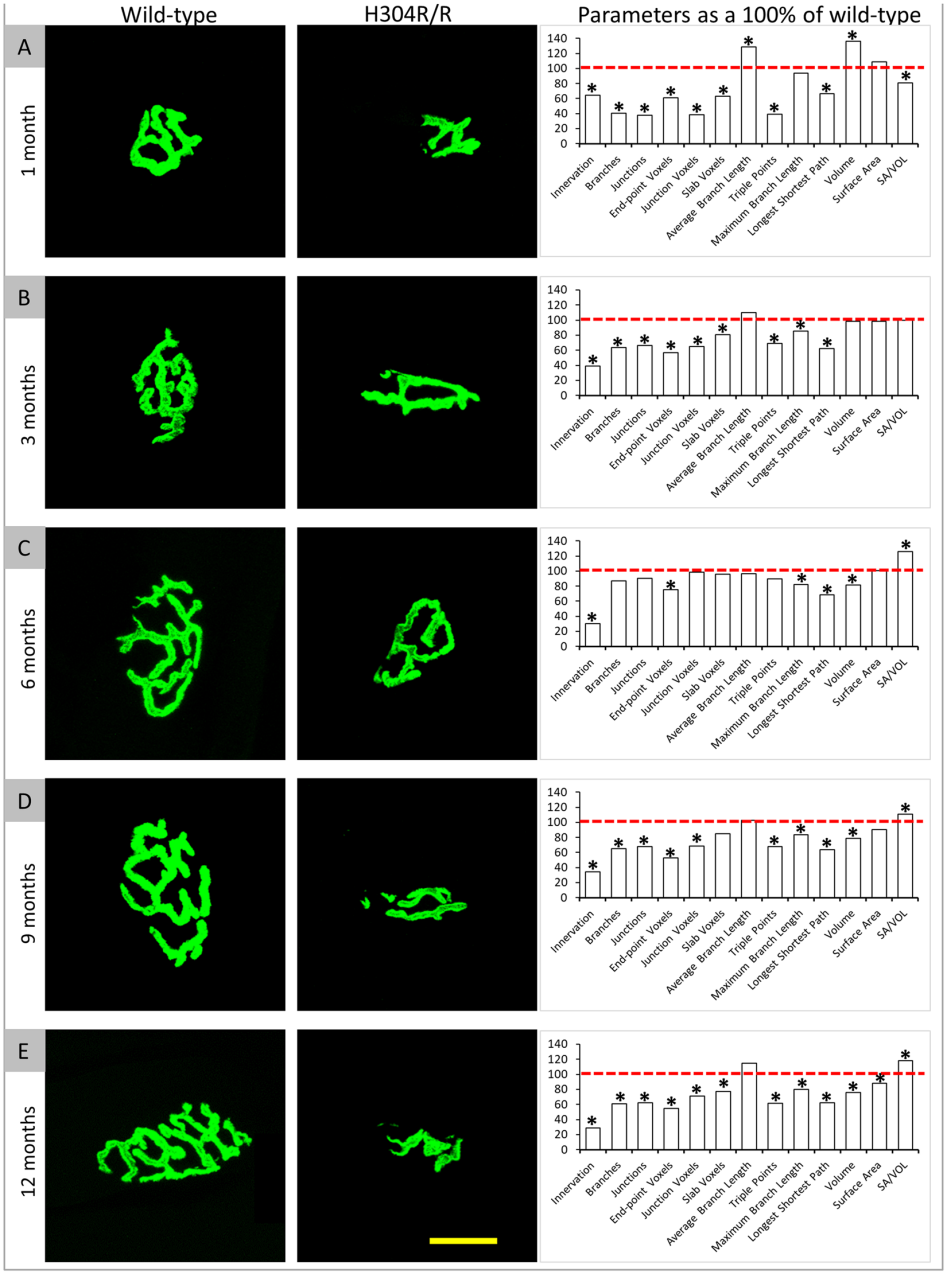
Confocal images of representative NMJs showing the acetylcholine receptors (AChR) stained with alpha-bungarotoxin (green) at different time points. 1 month (**A**), 3 months (**B**), 6 months (**C**), 9 months (**D**), and 12 months (**E**). Graphs show thirteen parameters that were examined for the NMJ architecture in H304R/R mice (white bars) as the percentage of the wild type value. The red dotted line is the wild type data normalized at 100. Statistical analysis was carried out using the Welch’s *t*-test (two-tailed distribution, *p < 0.05). The wild type data was generated and previously reported in Sabblah *et al*.^5^.

We previously showed that heterozygous mice bearing a mutation in the dynein heavy chain (H304R/+) show neuromuscular NMJ dysmorphology^5^. In this study we extended those studies to examine the NMJs of H304R/R homozygous mice carrying two copies of the H304R mutation. We found that the NMJs from gastrocnemius muscles of homozygous H304R/R mice displayed multiple severe abnormalities compared to wild type and heterozygous H304R/+ mice. We proceeded with a longitudinal assessment of NMJ architecture at 1 month, 3 months, 6 months, 9 months, and 12 months employing complexity parameters previously described to determine NMJ complexity^5^. One notable feature following the quantification of NMJ architecture in homozygous H304R/R mice is the presence of defects at all the time points examined (Fig. 8). (Suppl. Table 6) This was not the case with heterozygous H304R/+ mice where no statistically significant differences in NMJ complexity were observed at 3 months when compared to wild type^5^. The number of parameters altered and the extent of the abnormalities were much larger than we saw for H3046/ + NMJs in our previous study. This trend is consistent with the results of our motor skills behavior analyses and gait analyses described earlier.

### Homozygous H304R/R mice have an altered tail flick response

**Figure 9:**
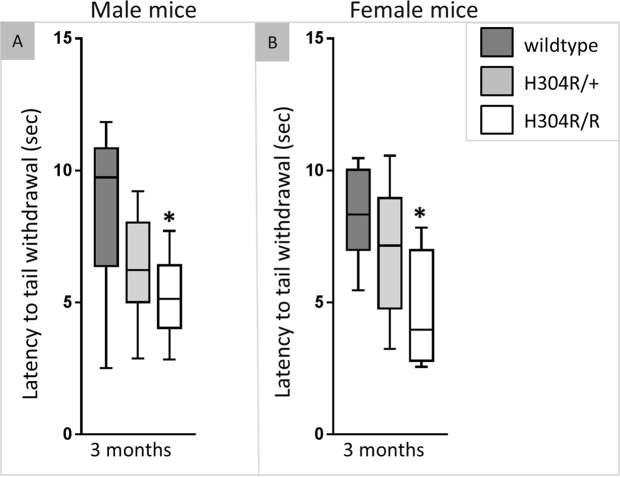
Tail flick response test in wild type, H304R/+, and H304R/R mutant mice at 3 months of age. The graph shows the latency of tail withdrawal (seconds) to pain stimulus in male (**A**) and female (**B**) mice. The box plot represents the 25^th^ to 75^th^ percentile of the data points with a middle line at the median and the whiskers ranging from the smallest to the largest value. The tail flick data was compared between the wild type (in dark gray), H304R/+ (in light gray), and H304R/R (in white) mice using the Kruskal-Wallis ANOVA test (*p < 0.05).

Tail flick tests were performed to assess the sensory response of H304R mice to a pain stimulus. Male heterozygous H304R/+ and homozygous H304R/R mice exhibited a quicker tail withdrawal on the tail flick test as compared to the wild type mice (Fig. 9; Suppl. Table 7). However, the tail flick response was statistically significant only for the male homozygous H304R/R mice as compared to the wild-type mice (*p* < 0.05). We also tested the female heterozygous H304R/+ and homozygous H304R/R mice on the tail flick test. Female H304R/+ and H304R/R mice exhibited a reduced time to tail withdrawal as compared to the wild type mice. However, this difference was statistically significant only in the H304R/R females (*p* < 0.05). Overall, both male and female H304R/R mice exhibited significantly quicker tail withdrawal on the tail flick test at 3 months of age, suggesting that they might have hypersensitivity to the pain stimulus.

## Discussion

To gain a better understanding of the onset and progression of axonal CMT2 as well as the role of cytoplasmic dynein in disease, we characterized a homozygous mouse line carrying the autosomal dominant H304R mutation in the *Dync1h1* DHC gene. We examined both male and female H304R/R mice in a 12-month longitudinal study to determine the impact of the H304R dynein DHC mutation has as a homozygous allele. The initial and one of the most important findings was that the homozygous H304R/R mice not only survived after birth, but also lived a normal lifespan. This is in stark contrast to the mutant DHC homozygous *Loa/Loa*, *Cra/Cra*, and *Swl/Swl* mice (which do not correspond to human disease alleles) that died due to embryonic or neonatal lethality. This illustrates that the mechanism of action of the H304R allele must be distinct from that of the *Loa*, *Cra*, and *Swl* alleles. Furthermore, our study of an autosomal dominant CMT2 mutation in a model system where both alleles are mutated (the homozygous condition) is an important addition now that there is a report of human CMT2 patients carrying similarly homozygous autosomal dominant mutations^12^. Therefore the combination of heterozygous H304R/+ and homozygous H304R/R mice, with differential severity of symptoms, provides a critical tool that can be utilized to study the function and dysfunction of dynein in various neuropathies.

In this work, we established that the homozygous H304R/R mice have severe defects in their motor skills behavior. Both male and female H304R/R mice exhibited an atypical tail suspension reflex of clenching their hind limbs (Fig. 1), a significant reduction in their hind limb grip strengths (Fig. 2), and motor coordination deficits in the rotarod assay (Fig. 3). These findings with H304R/R mice were mostly similar with the observations of atypical tail suspension reflex, reductions in the hind limb strength, and diminished rotarod performance in previously published heterozygous *Loa*/+, *Cra*/+, and *Swl*/+ mice^6–11^. In contrast, our previous study of heterozygous H304R/+ mice showed phenotypes that were generally milder than those seen in *Loa*/+, *Cra*/+, and *Swl*/+ mice^5^. Therefore, there is a clear severity difference between H304R, *Loa*, *Cra*, and *Swl* alleles with heterozygous H304R/+ mice generally showing more subtle phenotypes than heterozygous *Loa*/+, *Cra*/+, and *Swl*/+ mice. One potential limitation of the current study is that the behavioral data for wild-type and heterozygous H304R/+ mice was collected at an earlier time and published^5^ prior to the collection of the behavioral data for homozygous H304R/R mice. All other analyses presented here utilize data that was collected at the same time for the three genotypes of mice.

Likewise, the neonatal lethality phenotype observed in homozygous *Loa/Loa*, *Cra/Cra*, and *Swl/Swl* mice does not occur in homozygous H304R/R mice. There are indications from this work, and from our earlier study^5^, that there may be some developmental aspects to the phenotypes we see for H304R/+ and H304R/R mice. Future work that focuses upon early development may provide some key insights to how the phenotypes we observe in adulthood may come about.

We observed an interesting pattern in the degree that the sex of animals plays in the phenotypes associated with H304R alleles. In our previous study with heterozygous H304R/+ mice, we saw a clear difference in the severity of locomotor phenotypes between male and female mice^5^. Female H304R/+ mice showed little to no difference in phenotypes compared to wild type mice in the majority of time points and conditions examined. Male H304R/+ mice exhibited altered phenotypes in a wider range of time points in our tail suspension, grip strength, and rotorod locomotor assays. In contrast, both male and female homozygous H304R/R mice showed similar deficiencies in their locomotor behavioral phenotypes among assays and across time points (Figs 1–3). It may be that the increased severity of phenotypes in homozygous animals masks the more subtle difference that sex can play in the presentation of disease characteristics.

Upon initial observation, H304R/R mice exhibited an obvious high stepping gait when walking at a natural speed, and H304R/+ showed a less pronounced but still noticeable high stepping gait. Heterozygous *Loa*/+ and *Swl*/+ mice also display altered gait phenotypes but are described as waddling, wobbly, or unsteady^9,20^. We performed quantitative gait analysis at a forced constant velocity on the H304R/+ and H304R/R mice and identified several parameters of gait that were affected by the H304R DHC mutation (Figs 4 and 5). The affected parameters in H304R/+ mice included increased time spent in the swing phase in H304R/+ mice, and in H304R/R mice included shorter stride length, increased stride frequency, reduced propulsion, and altered paw angle. *Loa*/+ mice exhibited a wider hind limb stance with unaffected swing and propulsion phases when quantitatively analyzed^20^, inconsistent with our observations of the H304R/+ and H304R/R mice. However, the gait of *Loa*/+ mice was analyzed at a natural pace rather than a forced constant speed. Analyzing the H304R/+ and H304R/R mice at a natural pace may help to identify more pronounced variations in the gait of the mice at additional time points. Because the H304R/R mouse model exhibits a high stepping gait, decreased stride length, and reduced propulsion, it is the first mouse model with a DHC mutation to recapitulate the gait phenotypes observed in humans with CMT^13,21^. Thus, H304R/R mice will play an important role in understanding CMT onset and progression.

We have shown here in this study and in our previous work^5^ that the neuromuscular junction is a pathological target in CMT2O disease. The abnormalities in the NMJ are obvious in both heterozygous H304R/+ and homozygous H304R/R models, with the deformities worsening in the homozygotes. It is not hard to see how defects in NMJ structure and function could lead to the loss of muscle strength and coordination that are hallmark symptoms of CMT2 disease. A legitimate question that arises from an examination of our data is how does altered dynein function affect NMJ architecture? It is known that dynein retrograde transport of essential cargoes in axons is important in many neuronal processes^22^. We can therefore hypothesize that NMJ defects in H304R mouse models may be due to dynein dysfunction in the motor neurons that innervate the axon. There is evidence of that in our study where we saw reduced innervation and a concomitant increase in NMJ dysmorphology. An alteration in dynein activity in the muscle could also be a contributory factor to the phenotypes we see. It was discovered that disrupting dynein function alters the components of the neuromuscular synapse in primary myoblasts from mice^23^. More studies will be required to tease apart these or other mechanisms for how autosomal dominant mutations in dynein cause the severe defects we see in the NMJ.

Initial sensory assessment with the tail flick test revealed that the H304R/R mice have a significantly reduced time to withdraw their tails from a thermal pain stimulus (Fig. 6). These observations in H304R/R mice indicate a hypersensitivity to the pain stimulus that is often seen in the neuropathic pain rodent models^24,25^. This is also consistent with observations that some CMT2O patients report having significant neuropathic pain and paresthesia (tingling sensation) in their lower legs^3^. In relation to other mutant DHC mouse models, *Loa*/+, *Cra*/+ and *Swl*/+ mice display peripheral sensory deficits^9,26^. *Loa*/+ and *Swl*/+ mice had thinner dorsal root sizes, thinner diameters of sciatic nerves, loss of neurons in the lumbar DRG, and abnormal peripheral proprioception as evident by the absence of H-reflex^9^. *Swl*/+ mice showed no difference in the tail flick latency test as compared to the wild-type control animals; *Loa*/+ and *Cra*/+ mice have not been reported. The *Cra*/+ mice presented with less severe sensory deficits than the *Loa*/+ and *Swl*/+ mice, and showed thinner dorsal roots and degeneration of large caliber axons in its dorsal root^26^. It would be interesting for future studies to examine the sensory anatomy of H304R/+ and H304R/R mice to understand the effect of the H304R dynein mutation in sensory neuropathies such as CMT2O.

## Methods

### Colony breeding & maintenance

All experiments with mice were approved by the University of Central Florida IACUC committee, and followed all relevant guidelines and regulations. The H304R mouse model was generated using gene targeted knock-in method as described previously^5^. The founder H304R mice were further bred to generate a colony of mixed genotypes. Male and female heterozygous H304R/+ mice were cross-bred to yield wild-type, heterozygous H304R/+ and homozygous H304R/R littermate pups for this study. Each mouse genotype was confirmed via a PCR-based genotyping method^5^. Western blot analyses of dynein protein levels was performed as described previously ^5^ The H304R mice were identified throughout the study by a unique tail tattoo number and/or an ear punch. All mice were housed in an environment-controlled vivarium facility with access to food and water *ad libitum*.

### Mouse behavior tests

The researchers were blinded to the animals’ genotype while administering all tests. The mice were tested at various time points in multiple motor skills assessments. These test procedures for homozygous were performed as described previously^5^. Data from each test mice were respectively averaged, binned per 3-month time intervals (3, 6, 9 and 12 months), and compared between genotypes using statistical methods (Welch’s *t*-test, two-tailed distribution and *p* < 0.05). The data for the wild-type and H304R/+ mice were collected in our previous paper^5^ and compared to the data for the homozygous H304R/R presented here.

### Gait analysis

Mice were tested on the DigiGait treadmill device (Mouse Specifics) at multiple time points from 1 to 12 months. The DigiGait test was performed by placing the mouse inside the device’s transparent chamber. The transparent belt was set to a speed of 28 cm/s and was started upon the test mouse facing the proper direction. Each test mouse was recorded from the ventral angle using the DigiGait Imager software. Video recordings were cut down to 3–7 seconds and were processed using the DigiGait analysis software, which allows the paws to be digitally detected, producing a graph of the stride for each paw. The resulting stride graphs were compared to the corresponding video recording, and any discrepancies were addressed using the appropriate editing functions of the analysis software. The analysis software was then used to quantify the stride information from the stride graphs of each paw. Each test produced two hind limb data sets for the test mouse. The mean and standard deviation of the data were determined for each parameter. The H304R/+ and H304R/R data were statistically compared to the wild-type data using one-way ANOVA with Tukey’s multiple comparison post-test, *p* < 0.05.

### Immunohistochemistry and image analyses

Cryosections were prepared, stained, and analyzed as described in^5^ with the following addition. Synaptophysin staining was performed overnight at 4 °C with a polyclonal antibody (ABCAM). The percentage of synaptophysin occupancy was determined from the area enclosed by synaptophysin staining, divided by the area of acetylcholine receptors (stained by alpha-bungarotoxin).

### Sensory test

A tail flick instrument (PanLab Harvard Apparatus LE7106) was used to perform a sensory phenotype test. Each test mouse was gently held in a plastic restrainer and allowed to acclimate in the restrainer for 2 minutes. Then the mouse’s tail was exposed to a pain stimulus that was a thermal beam of light at a moderate intensity (50 on a scale of 99). The instrument recorded the latency to tail withdrawal, i.e. the time a test mouse took to remove or “flick” away its tail from the thermal beam path. The response time of tail flick reflex was automatically recorded and collected from the test mice at 3 months of age. The tail flick data was then averaged and compared between genotypes using the Kruskal-Wallis test (p < 0.05) due to non-normal distribution of the data.

## Supplementary information

Supplemental data
