# Supplementary material for: Mice with an autosomal dominant Charcot-Marie-Tooth type 2O disease mutation in both dynein alleles display severe moto-sensory phenotypes
Source: Sci Rep. 2019 Aug 19;9:11979. doi: 10.1038/s41598-019-48431-7 (PMC6700207; doi:10.1038/s41598-019-48431-7)
Supplement: Supplementary file 1 — Supplemental data [file 41598_2019_48431_MOESM1_ESM.pdf]

Supplemental information for

Mice with an autosomal dominant Charcot-Marie-Tooth type 2O disease mutation in both dynein alleles display severe moto-sensory phenotypes

Swaran Nandini, Jami L. Conley Calderon, Thywill T. Sabblah, Rachal Love, Linda E. King, and Stephen J. King*

* corresponding author

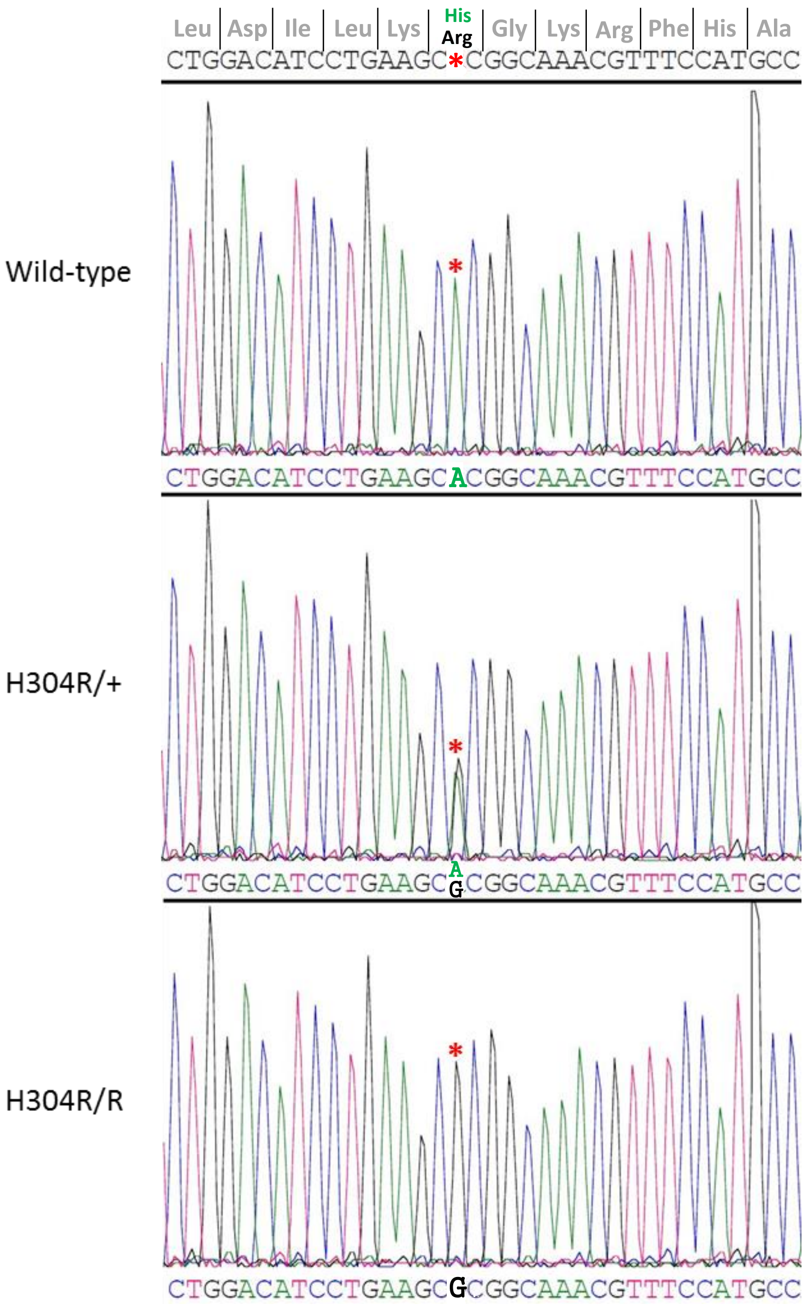

Supplemental Figure 1. Sanger sequencing data of wild-type, H304R/+, and H304R/R mice. The red asterisk indicates the position of the point mutation at nucleotide 911 of the coding sequence. Adenine (A) at the indicated position encodes histidine (H) at amino acid 304. Guanine (G) at the indicated position encodes arginine (R) at amino acid 304.

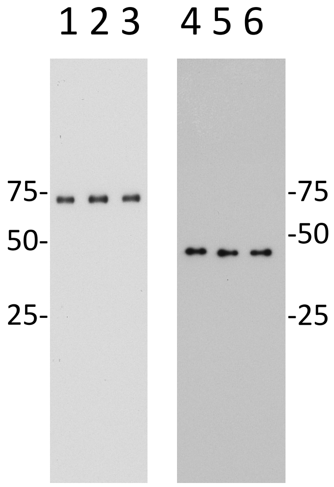

Supplemental Figure 2. Dynein complex protein levels in wild type, H304R/+ and H304R/R brain tissue. Brain tissue high speed supernatants were run on identical gels, the proteins were blotted onto polyvinyldiflouride membrane, and the Western blots were probed with antibodies for the dynein intermediate chain (left blot) or glyceraldehyde-3-phosphate dehydrogenase (right blot, loading control). Identical amounts of high speed brain tissue supernatant samples were loaded as follows: wild-type (lanes 1 and 4), H304R/+ (lanes 2 and 5), and H304R/R (lanes 3 and 6). Quantification of band intensities determined that there were no statistically significant difference in dynein intermediate chain levels between wild type, H304R/+ and H304R/R samples. (Students *t*-test, two tailed distribution, $p > 0.10$ for all comparisons).

Supplemental Table 1. Tail suspension reflex – Percentage of mice showing atypical tail suspension reflex (%), number of animals (n) and the Fisher's exact test *p*-values for the wild-type, heterozygous (H304R/+) and homozygous (H304R/R) mice.

Tail suspension reflex	Time point (age)	Percentage of mice showing atypical tail suspension reflex		Number of animals (n)		*p*-value (Fisher's exact test)
		Wild-type	H304R/R	Wild-type	H304R/R	Wild-type vs H304R/R
Male mice	3 months	7.5%	61.7%	34	26	**<0.0001**
	6 months	5.8%	76.6%	46	20	**<0.0001**
	9 months	9.6%	74.7%	29	20	**<0.0001**
	12 months	12.2%	79.6%	40	18	**<0.0001**
Female mice	3 months	10.0%	66.3%	9	27	**<0.0001**
	6 months	8.0%	62.6%	35	20	**<0.0001**
	9 months	11.3%	70.9%	54	19	**<0.0001**
	12 months	11.3%	70.7%	56	19	**<0.0001**

Supplemental Table 2. Male behavior mice –Mean values (± standard deviation), number of animals (n), and the Welch's *t*-test *p*-values for the male wild-type, heterozygous (H304R/+) and homozygous (H304R/R) mice.

Tests (male mice)	Time point	Mean ± std. dev		Number of animals (n)		*p*-value (Welch's *t*-test)
		Wild-type	H304R/R	Wild-type	H304R/R	Wild-type vs H304R/R
All limbs grip strength (grams)	3 months	212.5 ± 23.2	162.0 ± 46.3	50	26	**<0.0001**
	6 months	225.1 ± 30.8	162.9 ± 31.4	62	20	**<0.0001**
	9 months	230.6 ± 37.9	162.2 ± 38.6	41	20	**<0.0001**
	12 months	244.5 ± 32.1	167.0 ± 31.3	40	18	**<0.0001**
Front limbs grip strength (grams)	3 months	99.2 ± 12.9	96.0 ± 23.6	50	26	0.9995
	6 months	88.8 ± 14.3	84.6 ± 21.7	62	20	0.9970
	9 months	89.8 ± 12.9	85.8 ± 18.8	41	20	0.9987
	12 months	92.0 ± 11.9	84.1 ± 20.2	40	18	0.7910
Rotarod (seconds)	3 months	78.8 ± 40.9	25.0 ± 9.6	51	26	**<0.0001**
	6 months	95.5 ± 49.1	22.4 ± 9.7	48	20	**<0.0001**
	9 months	101.9 ± 52.9	17.8 ± 9.6	28	20	**<0.0001**
	12 months	100.7 ± 48.6	18.8 ± 9.7	20	18	**<0.0001**

Supplemental Table 3. Female behavior mice - Mean values (± standard deviation), number of animals (n), and the Welch's *t*-test *p*-values for the female wild-type, heterozygous (H304R/+) and homozygous (H304R/R) mice.

Test (female mice)	Time point	Mean ± std. dev		Number of animals (n)		*p*-value (Welch's *t*-test)
		Wild-type	H304R/R	Wild-type	H304R/R	Wild-type vs H304R/R
All limbs grip strength (grams)	3 months	175.5 ± 24.0	154.5 ± 23.0	51	27	**0.0004**
	6 months	192.9 ± 15.9	164.9 ± 28.5	56	20	**0.0004**
	9 months	209.8 ± 18.8	161.0 ± 29.5	55	19	**<0.0001**
	12 months	222.2 ± 22.2	173.2 ± 39.2	54	19	**<0.0001**
Front limbs grip strength (grams)	3 months	91.0 ± 12.5	91.5 ± 15.5	51	27	>0.9999
	6 months	81.5 ± 9.8	81.7 ± 17.0	56	20	>0.9999
	9 months	81.3 ± 11.8	81.6 ± 17.9	55	19	>0.9999
	12 months	85.9 ± 13.4	86.5 ± 21.1	54	19	>0.9999
Rotarod (seconds)	3 months	103.6 ± 47.6	28.1 ± 9.1	51	27	**<0.0001**
	6 months	113.7 ± 54.9	30.8 ± 18.7	51	20	**<0.0001**
	9 months	106.8 ± 61.4	23.9 ± 12.0	51	19	**<0.0001**
	12 months	98.8 ± 62.6	23.2 ± 9.8	50	19	**<0.0001**

Supplemental Table 4. DigiGait Analysis—Mean values (± standard deviation) for the various hind limb gait parameters measured, number of mice tested (N), and number of data points analyzed (n). Each mouse produced two data points for each parameter, one for each hind limb.

Sex	Time point	Genotype	n	N	% Swing of stride (%)	% Propel of stance (%)	Stride length (cm)	Stride frequency (steps/s)	Stance Width (cm)	Absolute Paw Angle (degrees)
Males	1 month	Wild-type	20	10	33.44 ± 3.32	78.42 ± 8.07	5.42 ± 0.54	5.23 ± 0.46	2.36 ± 0.15	13.38 ± 4.70
		H304R/+	20	10	36.75 ± 2.70	76.50 ± 7.29	5.75 ± 0.89	5.06 ± 0.79	2.52 ± 0.43	14.44 ± 4.03
		H304R/R	16	8	35.04 ± 3.03	75.64 ± 10.38	4.81 ± 0.47	5.16 ± 1.01	2.46 ± 0.23	18.58 ± 5.44
	3 months	Wild-type	24	12	35.30 ± 3.05	81.16 ± 8.22	6.38 ± 0.38	4.48 ± 0.27	2.68 ± 0.27	10.77 ± 4.01
		H304R/+	24	12	36.53 ± 2.91	80.24 ± 8.69	6.51 ± 0.72	4.42 ± 0.52	2.68 ± 0.22	11.26 ± 6.44
		H304R/R	12	6	37.37 ± 2.33	73.93 ± 5.74	5.87 ± 0.19	4.86 ± 0.16	2.95 ± 0.21	18.93 ± 7.10
	6 months	Wild-type	16	8	35.44 ± 2.45	86.55 ± 7.03	6.55 ± 0.17	4.33 ± 0.11	2.90 ± 0.26	11.19 ± 2.61
		H304R/+	14	7	35.31 ± 2.69	80.93 ± 6.43	6.56 ± 0.37	4.34 ± 0.25	2.91 ± 0.20	14.56 ± 1.60
		H304R/R	16	8	34.68 ± 2.65	79.76 ± 9.63	6.14 ± 0.48	4.66 ± 0.37	2.81 ± 0.30	18.31 ± 9.12
	9 months	Wild-type	26	13	33.98 ± 3.13	85.04 ± 5.93	6.54 ± 0.32	4.35 ± 0.22	2.85 ± 0.32	7.70 ± 5.63
		H304R/+	18	9	35.76 ± 2.93	82.83 ± 7.58	6.71 ± 0.72	4.29 ± 0.49	2.91 ± 0.24	13.94 ± 2.93
		H304R/R	22	11	33.83 ± 2.89	81.63 ± 8.35	6.35 ± 0.52	4.51 ± 0.36	2.83 ± 0.25	13.67 ± 7.97
	12 months	Wild-type	16	8	33.43 ± 2.94	85.76 ± 4.42	6.60 ± 0.54	4.34 ± 0.34	2.80 ± 0.36	8.39 ± 5.77
		H304R/+	16	8	34.09 ± 3.22	82.61 ± 6.11	6.42 ± 0.41	4.48 ± 0.33	2.79 ± 0.29	13.45 ± 2.66
		H304R/R	14	7	33.46 ± 2.49	81.22 ± 6.37	6.05 ± 0.21	4.71 ± 0.20	2.79 ± 0.32	13.69 ± 7.29
Females	1 month	Wild-type	22	11	33.41 ± 2.67	73.76 ± 8.69	5.49 ± 0.64	5.27 ± 0.58	2.48 ± 0.24	14.98 ± 3.69
		H304R/+	24	12	35.70 ± 3.44	77.53 ± 7.20	5.38 ± 0.64	5.38 ± 0.60	2.53 ± 0.32	13.78 ± 4.62
		H304R/R	14	7	35.79 ± 7.52	69.86 ± 9.93	5.01 ± 0.55	5.78 ± 0.67	2.51 ± 0.23	22.04 ± 5.99
	3 months	Wild-type	26	13	36.32 ± 3.85	80.08 ± 5.59	6.68 ± 0.40	4.25 ± 0.26	2.86 ± 0.30	13.38 ± 4.61
		H304R/+	22	11	37.00 ± 2.66	78.23 ± 9.36	6.62 ± 0.41	4.28 ± 0.26	2.63 ± 0.31	10.58 ± 4.18
		H304R/R	16	8	37.75 ± 3.02	73.75 ± 6.91	5.91 ± 0.83	4.91 ± 0.66	3.14 ± 0.29	24.52 ± 8.89
	6 months	Wild-type	14	7	34.91 ± 2.36	82.16 ± 7.09	6.82 ± 0.42	4.20 ± 0.27	2.90 ± 0.31	13.38 ± 5.70
		H304R/+	20	10	35.76 ± 3.17	82.58 ± 7.92	6.48 ± 1.17	4.65 ± 1.60	2.76 ± 0.20	10.87 ± 5.00
		H304R/R	10	5	37.56 ± 1.94	79.05 ± 8.80	6.52 ± 0.73	4.44 ± 0.51	3.26 ± 0.46	17.75 ± 11.05
	9 months	Wild-type	10	5	33.68 ± 4.03	77.61 ± 3.80	6.67 ± 0.22	4.25 ± 0.14	3.06 ± 0.32	12.55 ± 3.14
		H304R/+	12	6	35.80 ± 1.48	78.95 ± 3.67	6.59 ± 0.30	4.33 ± 0.21	2.90 ± 0.21	12.66 ± 5.34
		H304R/R	8	4	36.09 ± 1.72	79.71 ± 7.18	6.55 ± 0.63	4.36 ± 0.40	3.00 ± 0.28	12.08 ± 8.22
	12 months	Wild-type	16	8	32.47 ± 2.72	81.12 ± 6.10	6.58 ± 0.43	4.35 ± 0.32	2.93 ± 0.21	9.58 ± 5.38
		H304R/+	18	9	36.38 ± 3.25	82.30 ± 6.10	6.83 ± 0.51	4.17 ± 0.30	2.76 ± 0.21	8.08 ± 4.06
		H304R/R	10	5	34.63 ± 1.64	78.12 ± 11.81	6.87 ± 0.94	4.18 ± 0.56	3.00 ± 0.23	9.75 ± 8.59

Supplemental Table 5. Percentage of synapse occupancy—Mean values (± standard deviation), number of animals (N), number of samples tested (n), and the one-way ANOVA test *p*-values (with Tukey's multiple comparison) for the male wild-type, heterozygous (H304R/+) and homozygous (H304R/R) mice.

Timepoint	Genotype	N	n	Mean ± std dev (percent)	*p value* vs wild-type
**1 Month**	**Wild-type**	3	42	30.81 ± 20.92	-
	**H304R/+**	3	44	21.75 ± 23.01	0.0982
	**H304R/R**	3	53	15.91 ± 16.9	**0.0014**
**3 Months**	**Wild-type**	3	78	29.53 ± 24.40	-
	**H304R/+**	3	83	21.11 ± 20.17	0.0504
	**H304R/R**	3	96	20.90 ± 23.27	**0.0350**
**6 Months**	**Wild-type**	3	51	40.06 ± 16.79	-
	**H304R/+**	3	52	23.91 ± 21.64	**<0.0001**
	**H304R/R**	3	53	12.17 ± 16.42	**<0.0001**
**9 Months**	**Wild-type**	3	64	33.11 ± 16.73	-
	**H304R/+**	3	62	21.43 ± 20.65	**0.0071**
	**H304R/R**	3	71	15.73 ± 15.75	**<0.0001**
**12 Months**	**Wild-type**	3	69	33.56 ± 20.62	-
	**H304R/+**	3	74	21.51 ± 17.92	**0.0005**
	**H304R/R**	3	75	16.11 ± 17.91	**<0.0001**

Supplemental Table 6. NMJ analysis—Mean values (± standard deviation) for the various NMJ parameters analyzed, number of NMJs studied (n), number of mice from which tissue was analyzed (N), and the Welch's *t*-test *p*-values for the male wild-type and homozygous (H304R/R) mice.

Timepoint	Genotype	*n*	*N*	% of NMJ Innervated	Branches	Junctions	End-point Voxels	Junction Voxels	Slab Voxels	Average Branch Length	Triple Points	Maximum Branch Length	Longest Shortest Path	Volume	Surface Area	SA / VOL
1 month	wild-type	67	5	95.4%	27.0 ± 11.9	15.3 ± 7.1	7.1 ± 2.8	29.2 ± 14.6	353.4 ± 140.5	6.1 ± 1.4	14.4 ± 5.8	19.6 ± 5.8	56.6 ±15.8	687.4 ±253.3	1523.5 ± 476.6	2.3 ± 0.3
	H304R/R	56	4	64.3%	11.0 ± 6.7	5.8 ± 4.1	4.3 ± 2.0	11.2 ± 7.6	222.1 ± 101.0	7.9 ± 4.8	5.6 ± 4.0	18.4 ± 6.6	37.7 ± 11.9	933.2 ± 293.2	1659.1 ± 428.4	1.8 ± 0.3
	p value	-	-	-	**<0.0001**	**<0.0001**	**<0.0001**	**<0.0001**	**<0.0001**	**0.0087**	**<0.0001**	0.2912	**<0.0001**	**<0.0001**	0.0994	**<0.0001**
3 month	wild-type	67	6	93.9%	21.9 ± 9.1	11.6 ± 5.2	8.6 ± 3.1	21.8 ± 10.4	359.5 ± 111.6	7.6 ± 2.0	11.1 ± 4.9	22.3 ± 6.3	70.6 ± 20.3	1018.5 ± 400.3	1872.0 ± 561.38	1.9 ± 0.3
	H304R/R	95	6	38.8%	14.0 ± 8.6	7.7 ± 5.3	4.8 ± 2.2	14.1 ± 10.5	289.4 ± 134.4	8.3 ± 5.4	7.6 ± 5.3	19.0 ± 6.3	44.1 ± 12.2	1004.8 ± 419.0	1841.3 ± 622.6	1.9 ± 0.4
	p value	-	-	-	**<0.0001**	**<0.0001**	**<0.0001**	**<0.0001**	**<0.0001**	0.2498	**<0.0001**	**0.0013**	**<0.0001**	0.8336	0.7437	1
6 month	wild-type	98	5	88.8%	19.3 ± 9.5	10.1 ± 5.4	7.6 ± 3.4	20.1 ± 13.5	335.0 ± 137.0	8.2 ± 2.9	9.5 ± 4.9	25.1 ± 8.9	68.9 ± 23.3	1278.0 ± 630.4	2020.7 ± 790.6	1.7 ± 0.3
	H304R/R	87	5	30.5%	16.7 ± 11.2	9.1 ± 6.7	5.7 ± 2.7	19.8 ± 27.8	321.0 ± 150.0	8.0 ± 5.2	8.6 ± 6.3	20.4 ± 7.2	47.4 ± 15.1	1042.3 ± 468.4	2035.2 ± 728.5	2.1 ± 0.5
	p value	-	-	-	0.0926	0.2692	**<0.0001**	0.9272	0.5102	0.7241	0.2841	**<0.0001**	**<0.0001**	**0.0041**	0.8969	**<0.0001**
9 month	wild-type	63	5	86.2%	22.0 ± 11.5	11.4 ± 6.6	9.1 ± 3.6	22.7 ± 17.6	362.8 ± 363.3	8.2 ± 3.2	10.8 ± 6.2	25.3 ± 9.0	74.7 ± 24.6	1051.7 ± 670.3	1925.0 ± 922.0	2.1 ± 0.5
	H304R/R	75	5	34.5%	14.3 ± 7.7	7.8 ± 4.6	4.9 ± 2.2	15.5 ± 10.0	309.3 ± 130.1	8.4 ± 4.5	7.4 ± 4.3	21.1 ± 7.5	47.5 ± 13.8	828.4 ± 399.5	1739.5 ± 641.0	2.3 ± 0.6
	p value	-	-	-	**<0.0001**	**0.0004**	**<0.0001**	**0.0049**	0.2703	0.7615	**0.0004**	**0.0039**	**<0.0001**	**0.0224**	0.1809	**0.0345**
12 month	wild-type	118	6	94.9%	22.8 ± 11.3	11.7 ± 6.4	9.7 ± 4.0	19.9 ± 12.8	387.7 ± 155.7	8.0 ± 2.5	11.2 ± 6.0	25.9 ± 9.5	78.5 ± 24.4	1438.3 ± 718.8	2281.7 ± 907.3	1.7 ± 0.3
	H304R/R	88	6	28.7%	13.78 ± 8.3	7.3 ± 5.0	5.3 ± 2.4	14.1 ± 9.6	298.8 ± 140.2	9.1 ± 7.5	6.9 ± 4.8	20.6 ± 8.8	48.9 ± 14.5	1091.2 ± 546.7	2004.9 ± 811.0	2.0 ± 0.4
	p value	-	-	-	**<0.0001**	**<0.0001**	**<0.0001**	**0.0003**	**<0.0001**	0.1891	**<0.0001**	**<0.0001**	**<0.0001**	**<0.0001**	**0.0223**	**<0.0001**

Supplemental Table 7. Tail flick test – Average time of response (sec), number of animals (n), and the Kruskal-Wallis test *p*-values for the wild-type, heterozygous (H304R/+) and homozygous (H304R/R) mice.

Tail flick test	Time point (age)	Average time of response to the pain stimulus (seconds)			Number of animals (n)			*p*-value (Kruskal-Wallis test)		
		Wild-type	H304R/+	H304R/R	Wild-type	H304R/+	H304R/R	Wild-type vs H304R/+	Wild-type vs H304R/R	H304R/+ vs H304R/R
Male mice	1 month	8.13	7.28	4.46	3	13	2	0.9051	0.4203	0.4743
	3 months	8.64	6.40	5.21	7	18	6	0.0843	**0.0284**	0.5117
Female mice	1 month	7.60	7.77	7.86	5	9	4	0.9876	0.9796	0.9968
	3 months	8.43	7.04	4.59	9	12	4	0.3108	**0.0163**	0.1335
